# Biodefense Shield and Avian Influenza

**DOI:** 10.3201/eid1205.051480

**Published:** 2006-05

**Authors:** Ken Alibek, Ge Liu

**Affiliations:** * George Mason University, Manassas, Virginia, USA;; † AFG Biosolutions, Inc., Germantown, Maryland, USA

**Keywords:** biodefense, influenza A, flu pandemic, immunoprophylaxis, innate immunity, immunomodulators, interferons, pulmonary delivery, letter

**To the Editor:** In defending against avian influenza virus H5N1, the possibility of adopting treatments being developed for biodefense should not be overlooked. Biodefense medicine primarily concerns respiratory infections because bioweapons in their deadliest form disperse *Bacillus anthracis* and *Yersinia pestis*, the causes of anthrax and plague, and highly contagious viruses like smallpox, Ebola, and Marburg as aerosols. The National Institutes of Health and Department of Defense have funded developing novel biodefense medications designed to stimulate innate mucosal immunity by using interferons (IFNs) and interferon inducers. We suggest that studies begin immediately to explore the potential of IFNs to prevent infections and reduce deaths caused by avian influenza viruses in animal models and humans.

Modulating innate mucosal immunity is promising as a rapid-acting, broad-spectrum approach to combat bioterrorism (*1*). Innate immunity, the initial response to a pathogen, is potentially capable of eradicating infection. Even when the innate immune response cannot eliminate a virus, it may substantially reduce viral load, reduce pathology, facilitate clearing of the virus by the adaptive immune response, and slow the spread of infection (*1*). As biodefense medications, IFNs and IFN-inducers are under development for aerosolized delivery to the lungs (*2**,**3*). Conventional IFN administration by injection often results in low concentrations at target sites and high concentrations in circulation, which may cause serious side effects. Aerosolized delivery minimizes side effects and produces more rapid clinical responses. Inhaled IFNs have proven to be well tolerated and beneficial for rhinovirus infection (*4*) and pulmonary tuberculosis (*5*).

Medications being developed to prevent infections caused by viral bioweapons and other diseases include 1) Oral IFN-α or Alferon low dose oral (LDO) (Hemispherx Biopharma, Inc., Philadelphia, PA, USA); 2) inhalable IFN-γ (InterMune, Brisbane, CA, USA); 3) dsRNA [Poly (ICLC)] or Ampligen (Hemispherx Biopharma, Inc.); 4) ssRNA (Aldara and Resiquimod from 3M Pharmaceuticals, St. Paul, MN, USA); and 5) CpG7909 and CpG10101 oligonucleotides (Coley Pharmaceutical Group, Wellesley, MA, USA) (*2*). These drugs have either been approved by the Food and Drug Administration (FDA) (Aldara), are in clinical trials (Alferon LDO, inhalable IFN-γ, Resiquimod, CPG7909, and CpG10101), or at a preclinical stage of development (Ampligen). Aldara is approved for genital warts, actinic keratoses, and basal cell carcinoma. Others drugs are being tested for aerosolized delivery to modulate mucosal immunity of the respiratory tract. All could be expeditiously tested with inhalational or intranasal administration in H5N1 models with mice, ferrets, pigs, and monkeys.

IFN-α and IFN-γ work by binding their receptors and activating downstream antiviral pathways involving the dsRNA-dependent protein kinase (PKR), the 2´, 5´ oligoadenylate synthetase/RNase L, or the MxA protein. dsRNA, ssRNA, and CpG oligonucleotides are ligands for toll-like receptors (TLRs) and modulate antiviral immunity through TLR signaling pathways and IFN induction (*2*). At the cellular level inside the lungs, these drugs will enhance phagocytotic and cytolytic activity in alveolar macrophages.

Once infection is established, H5N1 resists the antiviral effects of IFNs and tumor necrosis factor-α (*6*). Resistance is associated with the nonstructural gene of H5N1 and may be 1 mechanism for H5N1's extraordinary virulence. Therefore, prophylactic use of IFNs and IFN-inducers is critical to combat H5N1. They may also be effective if administered immediately after infection.

IFN resistance also exists for other viral infections. For instance, poxviruses including vaccinia virus encode 2 proteins that interfere with RNaseL and PKR pathways and 2 soluble IFN receptors that interfere with IFN-induced antiviral pathways. Nevertheless, at least in animal models, pre-infection administration of exogenous IFN can reduce deaths and poxvirus viral load. In mice, intranasal administration of IFN-α and IFN-γ prevents lethal vaccinia infection (*3*). IFN-α, IFN-γ, and an IFN inducer, Poly (ICLC), protect mice infected with H1N1 influenza virus (*7*). Hence, we suggest that anti-H5N1 prophylaxis by IFN-stimulated innate mucosal immunity is a promising therapy worth immediate investigation in animal models.

A second mechanism proposed to explain H5N1 virulence is also IFN related. This is the "cytokine storm," as shown by elevated levels of proinflammatory cytokines including IFNs found in 2 patients who died of H5N1 infections (*8*). Cytokine storms can result in autoimmune reactions, tissue damage, or septic shock. High IFN doses for long periods may exacerbate autoimmunity. However, despite similar cytokine storms (*9*), some severe acute respiratory syndrome patients respond well to IFN therapy (*10*). Optimal formulation and regimen of IFN administration could be crucial to effective anti-H5N1 prophylaxis. In the interests of safety, we propose that initial prophylaxis studies use relatively low IFN doses for short periods (≈1–2 weeks).

It is unlikely that all of these drugs will effectively protect against H5N1. And a drug that is effective might not work for everyone; genetic polymorphism influences IFN response. However, FDA approval of even one of them might save many lives.
